# Why Do People Support Online Crowdfunding Charities? A Case Study From China

**DOI:** 10.3389/fpsyg.2021.582508

**Published:** 2021-02-26

**Authors:** Huifang Jiao, Lamei Qian, Tianzhuo Liu, Lijun Ma

**Affiliations:** ^1^Department of Management Science, Shenzhen University, Shenzhen, China; ^2^Department of Management Science, University of Science and Technology of China, Hefei, China

**Keywords:** charitable crowdfunding, intrinsic motivations, extrinsic motivations, social interaction, willingness to share, intention to donate

## Abstract

Whereas the effect of people’s motivations to give to traditional, off-line charities has been extensively investigated, their motivations to support online charitable crowdfunding projects are largely unexplored. The present study examines the influences of extrinsic motivations (such as reputation, the wish to signal a certain image; and reciprocity, the expectation on future return for their present contribution), intrinsic motivations (such as a sense of belonging, the feeling of belonging to an integral part of a positive community; joy of giving, the anticipated positive emotions experienced by helping others; altruism, intrinsic desire to help others without expectation of a return; and financial constraints, individuals’ personally felt financial stresses induced by donations), and social interactions (such as social influence, people’s perception of how their close acquaintances believe they should support the project; and social ties, the strength of the relationship between the individual and the project initiator) on intentions to support charitable crowdfunding behaviors, namely, the willingness to share (WTS) project information and the intention to donate (ITD) money. Hierarchical multiple regression analyses on self-reported survey data from 617 respondents in China reveal support for the hypotheses. The results show that intrinsic motivations and social ties are predictors for both supporting behaviors. Reputation and social influence motivate people to share projects, but have no significant effects on their ITD. Reciprocity is positively, and financial constraints are negatively, related to ITD but have no significant effects on WTS. These findings offer insights into the motivations driving individuals’ participation in charitable crowdfunding. The implications for both platforms and fundraisers are discussed.

## Introduction

### Charitable Crowdfunding

Crowdfunding aims to collect funds through the Internet for commercial or charitable purposes (Choy and Schlagwein, 2016). Internet-based crowdfunding platforms allow project initiators to reach large crowds publicly (Gerber et al., 2012). In general, a charitable crowdfunding project involves three parties: the project initiator, who launches a project to be funded; supporters,

whose donations and sharing support the project; and platforms that bring project initiators and supporters together (Liu et al., 2018). In China, charitable crowdfunding platforms, such as Shuidichou, Qingsong Chou, and Aixin Chou, provide opportunities for any initiator to launch a project and request a certain amount of money that needs to be raised within a fixed period. Launching a charitable crowdfunding project on Social Network Services (SNSs) is a fast and far-reaching way to broadcast information to a large pool of users and to build widespread support, which maximizes the chances of its success (Cecere et al., 2017). WeChat, one of China’s largest SNSs, serves as a fundraising channel that allows users to raise money for charitable purposes. For reference, the WeChat page of a specific project on the medical treatment crowdfunding platform Shuidichou has been attached in the Supplementary Figure 1.

Charitable crowdfunding has become an important vehicle for social participation in charity fundraising (Jian and Shin, 2015). An increasing number of people are becoming involved in charitable crowdfunding because of the wide reach of social networking. Researchers have shown an increased interest in the expanding charitable crowdfunding phenomenon and especially in the reasons that motivate people to support it (Choy and Schlagwein, 2016).

### Previous Research on Charitable Crowdfunding

Previous studies of the reasons why people support crowdfunding concentrate on the field of reward-based crowdfunding, which involves individuals contributing comparatively small amounts of money to projects in return for some kind of reward. Intrinsic and extrinsic motivations are the main factors that affect individuals’ decisions to invest in such projects (Ryu and Kim, 2016). Studies investigating the extrinsic motivations for such decisions focus mainly on the rewards and find them to be positively related to investment decisions (Zhang et al., 2019). However, in charitable crowdfunding, there are no tangible returns for donors, and economic rewards are unlikely to be the main motive. Intrinsic motivations, including interest, playfulness, helping others, enjoyment, and a sense of belonging, are fully discussed in the literature on reward-based crowdfunding, but empirical evidence based on surveys is rare (Zhang and Chen, 2019). Some studies offer contradictory findings about the relationship between intrinsic motivations and investment decisions in the case of different projects. For example, Bretschneider and Leimeister (2017) found that altruism is not significantly related to funding intention, but Ryu et al. (2020) offered contrary results. A possible explanation might be the different characteristics of the different projects examined. Altruism constitutes a significant driver for investors in projects with charitable characteristics.

Some studies have also examined the factors influencing the intention to donate (ITD) in charitable crowdfunding (almost all the quantitative studies of which we are aware are listed in Supplementary Table 1). Sura et al. (2017) and Li et al. (2018) studied the influences of external factors such as project and platform characteristics on donation intention, ignoring personal-level motivations. The studies of Liu et al. (2018), Wang et al. (2019), and Chen et al. (2019) focused on the effects of personal-level factors such as trust, empathy, identity, and norms on charitable crowdfunding behaviors, but the focus of these studies was limited, and many motivation variables were not considered. Thus, it is necessary to empirically explore the different types of motivations driving charitable crowdfunding behavior.

### Motivations and Self-Determination Theory

Motivations refer to the reasons or goals that give rise to an action; they are widely used to explain behavioral intentions in psychology and Information Science (Hung et al., 2011). The “intrinsic vs. extrinsic motivation” model highlighted by SDT (Deci and Ryan, 2000) has been identified as being helpful and useful in both the traditional charity and crowdfunding literature (Choy and Schlagwein, 2016). Intrinsic motivations are defined as the engagement of an individual in an activity for personal interest, as well as for fun and enjoyment; extrinsic motivations refer to the gains the individual expects to make from performing the activity; usually these are external rewards (Ryan and Deci, 2000). Individuals weigh both extrinsic factors (e.g., potential future rewards) as well as intrinsic factors (e.g., pure altruism and joy of help others) when considering their crowdfunding behavior (Allison et al., 2015). Prosocial behavior in traditional charity has been attributed to both intrinsic and extrinsic motivations (Cox et al., 2018).

Charitable crowdfunding denotes the intersection between traditional charity and online community behavior (Choy and Schlagwein, 2016). On the one hand, charitable crowdfunding can be framed as philanthropy (Liu et al., 2018). Hence, both intrinsic and extrinsic motivations identified in traditional charity are also considered to drive charitable crowdfunding behaviors.

The intrinsic motivations of altruism and joy of giving have been found to be the main motives for giving in traditional charity (Echazu and Nocetti, 2015). Altruism takes the form of unconditional kind acts, without any expectation of a return (Ferguson et al., 2012). People whose giving is motivated by pure altruism are driven by their intrinsic desire to help others, regardless of personal costs and benefits. The joy of giving is the positive, self-directed, emotional reaction experienced by helping others and is known as the warm glow feeling (Bekkers and Wiepking, 2011). People are intrinsically motivated to give by referring to the amount of joy they anticipated to experience from the helping behavior (Erlandsson et al., 2016). Pure altruism and the joy of giving (the warm glow feeling) are expected to drive charitable crowdfunding behaviors as well as traditional charitable giving. Financial constraints are people’s perceived financial stresses caused by donations and are important negative influences on their donations in traditional charity contexts (Konrath and Handy, 2018). Financial constraints fall within donors’ personal sphere and are classified as intrinsic motivations that are negatively related to giving behaviors.

Extrinsic motivations, such as reputation and reciprocity, have been considered to motivate prosocial behavior in traditional charity (Edlund et al., 2007). Reputation refers to the social consequences of donations for donors: people are motivated to donate to gain social recognition (Bekkers and Wiepking, 2011). Reciprocity refers to the gains that people expect to make in the form of future benefits from their present actions (Hung et al., 2011): people are motivated to donate by the expectation that they will benefit from these gains in the future (Konrath and Handy, 2018).

### The Inclusion of Social Interaction–Based Motives in Charitable Crowdfunding

Conversely, charitable crowdfunding behavior can also be framed as prosocial behavior in online virtual communities (Liu et al., 2018). The motivations for charitable crowdfunding behaviors differ from traditional charity because of the nature of the IT platforms on which they occur. The supporters in charitable crowdfunding primarily desire to gain non-monetary rather than monetary benefits (Zhang X. et al., 2017). This differs from traditional charity giving, by means of which people can benefit by gaining monetary rewards (Bekkers and Wiepking, 2011). Thus, some extrinsic motivations identified in traditional charity (e.g., monetary reward and tax credits) may not be the main drivers of charitable crowdfunding behavior. Charitable crowdfunding platforms allow donors to help people in the platforms’ virtual communities (Choy and Schlagwein, 2016). Thus, the motives that drive user participation in virtual communities should be emphasized in charitable crowdfunding. For example, a sense of belonging is a vital intrinsic motivation that encourages sustained online participation in virtual communities and needs to be considered in charitable crowdfunding behavior (Zhao et al., 2012). The proliferation of charitable crowdfunding projects mainly depends on personal social networks. Individuals may share the project with their contacts and ask their friends to support the project; such friends may then also share this project with their contacts and solicit donations. In this way, social interactions between the donor, third parties (the donor’s social neighbors), and the project initiator are stronger than with traditional charity. Potential donors’ acts of giving can be observed by third parties (other members of the community), who have stronger social bonds with the donor. “Peer pressure” from seeing that social neighbors have donated (social influence) motivates them also to donate. Stronger social ties with the initiator also motivate people to donate. Thus, we expect that the social interaction variables of social influence and social ties motivate people to support charitable crowdfunding endeavors. Therefore, a comprehensive understanding of the motivations that support charitable crowdfunding is essential. Such an understanding would enable platforms to attract more people to help each other and create mutually supportive and like-minded communities.

### Two Different Types of Pro-sociality in Charitable Crowdfunding

Furthermore, charitable crowdfunding supporting behaviors include not only donation behavior but also sharing behavior. Sharing project information via social media channels can further facilitate the success of a project (Li et al., 2017). Sharing project information on social networking sites (SNSs) means that a project can be broadcast to a wide audience via a known and trustworthy intermediary, which can help boost the number of viewers. Increased social media exposure will, in turn, impact project funding. Sharing behavior can attract many more potential donations to the project, but this relationship has not been investigated in previous studies. The motivations behind donation and sharing behaviors might not be the same; thus, this study intends to explore the motivations behind both behavioral intentions.

To do so, we explore (1) the effects of both extrinsic and intrinsic motivations, based on insights from traditional charity and virtual community literature, and (2) social interaction motivations, taking into consideration the social characteristics of charitable crowdfunding on both charitable crowdfunding behavioral intentions, i.e., the willingness to share (WTS) project information and the ITD money to the project.

## The Present Research

**FIGURE 1:**
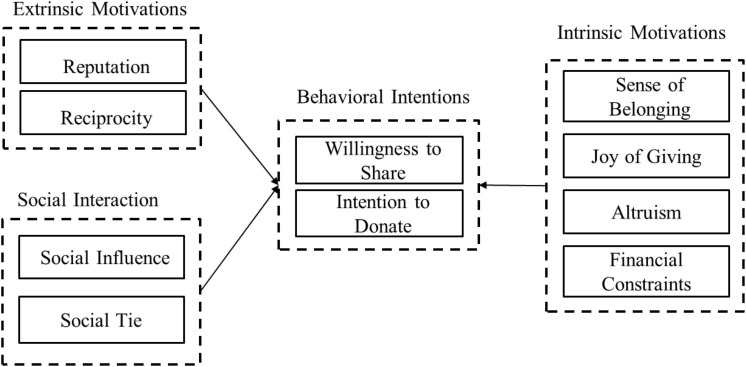
Research model.

Building on self-determination theory and previous research, we systematically investigated people’s motivations to support charitable crowdfunding behaviors (both WTS and ITD). To specify, using self-rated data, this study examines two extrinsic motivations (i.e., reputation and reciprocity), four intrinsic motivations (i.e., altruism, joy of giving, sense of belonging, and financial constraints), and two social interaction motivations (i.e., social influence and social ties) that may affect charitable crowdfunding behaviors, based on studies of traditional charities (Bekkers and Wiepking, 2011; Konrath and Handy, 2018), virtual communities (Zhang X. et al., 2017), and crowdfunding (Choy and Schlagwein, 2016; Jian and Shin, 2015). It focuses on reputation and reciprocity because they are the two non-monetary rewards often studied in philanthropy (Bretschneider and Leimeister, 2017). Unlike in traditional charity, external rewards are non-monetary in charitable crowdfunding. This research also focuses on four intrinsic motivations–namely, altruism, joy of giving, sense of belonging, and financial constraints–because these are important aspects of traditional charity, and we expect that they may also affect charitable crowdfunding behaviors. We also consider the social interaction factors of social influence and social ties because crowdfunding platforms change the strength of social ties and the social distances between the three parties (donors, social neighbors, and project initiators) involved in charitable projects. Previous studies have also found these two factors to have positive effects on charitable giving. For example, Jian and Shin (2015) found that social influence positively affects crowdfunding journaling behavior, and Sura et al. (2017) indicated that the online communication with the project initiator creates stronger social ties and increases people’s ITD. Figure 1 depicts our research model.

Intention to donate refers to the strength of individuals’ ITD money to charitable crowdfunding projects and was assessed with three items (e.g., “My willingness to donate money to the crowdfunding project is high”) (Liu et al., 2018). WTS refers to individuals’ WTS information about charitable crowdfunding projects and was measured with three items [e.g., “I would share this project on my social account (WeChat/Weibo)”] (Li et al., 2017).

## Hypotheses

### Extrinsic Motivations

#### Reputation

Reputation refers to “the general judgment or opinions about a person” and can help individuals achieve and maintain their status within a community (Zhang X. et al., 2017). Reputation motivation refers to individuals’ expectation on reputation feedback from contribution to charitable crowdfunding project. We developed a combination of multiple scales such as self-image concerns and social recognition to measure reputation by seven items (e.g., “I wish to signal a certain image of myself to others”) (Choy and Schlagwein, 2016). Charitable giving is viewed as a positive and prosocial behavior; thus, people who donate to charitable campaigns are highly regarded by their peers in a community, and they receive recognition and approval from others (Bekkers and Wiepking, 2011). Indeed, when given the choice, people often prefer making visible donations to signal their prosocial behavior and enhance their reputation (Andreoni and Petrie, 2004). More specifically, individuals tend to donate if they perceive that doing so will enhance their reputation. In the case of charitable crowdfunding, people are encouraged to share their donation experiences through personal social networks. It is much easier for people to signal a certain image of themselves to others via social media channels. When individuals feel that sharing project information or donating money to a project can elevate their reputation, they will be more inclined to support the project. Thus, the following hypotheses are proposed: when controlling for the other motivations,

**H1a.** Reputation is positively related to individuals’ WTS.
**H1b.** Reputation is positively related to individuals’ ITD.

#### Reciprocity

Reciprocity is a social norm that demands that people help those who have helped them and regulates interpersonal interactions (Hung et al., 2011). Reciprocity motivation in this research refers to individuals’ expectation on future return for their present contribution to charitable crowdfunding project and was measured by three items (e.g., “I expect that others will help me when I am in need for my contribution”) (Zhang X. et al., 2017). In a reciprocal society, people believe that everyone should try to repay that which another person has provided for them (Edlund et al., 2007). In online communities, reciprocity tends to be generalized, and people may expect future benefits not from the direct beneficiaries of their contributions but from other people who are implicated with the beneficiaries in a social exchange (Feng and Ye, 2016). Reciprocity helps build a sustainable feedback loop in online communities (Zhang X. et al., 2017). Research has also revealed that reciprocity significantly affects how much an individual contributes (Hung et al., 2011). Individuals share their donation experiences with others to help build reciprocal relationships. Thus, people who expect reciprocity will participate in more charitable crowdfunding projects of which they approve, and will have a higher ITD, to support charitable crowdfunding. Therefore, the following hypotheses are proposed: when controlling for the other motivations,

**H2a.** Reciprocity is positively related to individuals’ WTS.
**H2b.** Reciprocity is positively related to individuals’ ITD.

### Intrinsic Motivation

#### Sense of Belonging

A sense of belonging is defined as the experience of personal involvement in a community that makes people feel themselves to be an integral part of that community (Hagerty and Patusky, 1995). Sense of belonging in the present research was measured by asking how the subjects would feel when they actually supported the charitable crowdfunding project. Four items were used to assess their feelings (e.g., “I feel of belonging to a positive group/team or community”) following the suggestion by Choy and Schlagwein (2016). A sense of belonging is a fundamental motivation for people to form and maintain lasting, positive, and significant interpersonal relationships (Kim and Drumwright, 2016). This motivation encourages people to voluntarily participate in a virtual community (Zhao et al., 2012). People who value a sense of belonging to a community are more likely to participate in crowdfunding campaigns (Aitamurto, 2011). In charitable crowdfunding, donors are motivated to donate because they enjoy the feeling of belonging to a team or community. They also perceive the charitable crowdfunding crowd as positive and wish to be involved in a project with like-minded people (Choy and Schlagwein, 2016). This leads to the following hypotheses: when controlling for the other motivations,

**H3a.** Sense of belonging is positively related to individuals’ WTS.
**H3b.** Sense of belonging is positively related to individuals’ ITD.

#### Joy of Giving

The joy of giving (warm glow) is the positive psychological experience of the giver, arising from helping others (Bekkers and Wiepking, 2011). Joy of giving was measured by asking the subjects to rate how they would feel when they actually supported the charitable crowdfunding project. Four items were used to assess their feelings (e.g., “Contributing to the project makes me feel powerful”) (Choy and Schlagwein, 2016; Konrath and Handy, 2018). The reasons why people gain pleasurable experiences and positive emotional sensations from giving may be that such giving alleviates feelings of guilt, and that performing in line with a prosocial self-image and a social norm feels good (Bekkers and Wiepking, 2011). Experimental studies have shown that positive mood generally motivates giving (Dolinski et al., 2005). The positive relationship between donating behavior and the joy of giving has been proven by psychology literature, which suggests that donating promotes happiness and that, as the level of happiness increases, the likelihood of donating also increases (Ugur, 2018). Cecere et al. (2017) pointed out that a key motivation for people participating in charitable crowdfunding and making donations is that doing so gives them pleasure and good feelings from helping others. Hence, it is hypothesized that: when controlling for the other motivations,

**H4a.** Joy of giving is positively related to individuals’ WTS.
**H4b.** Joy of giving is positively related to individuals’ ITD.

#### Altruism

Altruism is a personal trait that embodies personal social responsibility and a sense of mission (Rodriguez-Ricardo et al., 2019). It is defined as an unconditional kindness toward others without expectation of a return (Fehr and Gachter, 2000). Altruism was measured by asking how much the subjects agree with the five statements about altruism trait (e.g., “I like helping other people even though it is not required”) (Liu et al., 2018). Altruistic people care about others’ well-being, they have pro-social attitudes, and they enjoy helping others with, in many cases, no expectation of any return. Altruism is included in the SDT model to explain why people are willing to give up their own resources to improve others’ welfare (Rodriguez-Ricardo et al., 2019). In traditional charity, it has long been recognized that altruistic impulses motivate charitable giving behaviors (Ribar and Wilhelm, 2002). In the context of crowdfunding, projects in need of help are favored by supporters, which implies that supporters are motivated by altruistic reasons (Burtch et al., 2013). Furthermore, interview-based studies on crowdfunding platforms also show altruism to be an important motivation for supporting projects (Gleasure and Feller, 2016). Therefore, we hypothesize the following: when controlling for the other motivations,

**H5a.** Altruism is positively related to individuals’ WTS.
**H5b.** Altruism is positively related to individuals’ ITD.

#### Financial Constraints

Constraints are factors that inhibit individuals’ ability to participate in activities; they include lack of money, time, skill, or interest (Filo et al., 2020). Financial constraints are defined as individuals’ personally felt financial stresses induced by donating money to charity (Konrath and Handy, 2018). Following Konrath and Handy (2018)’s research, financial constraints were measured by asking the subjects to rate how they would feel when they actually supported the charitable crowdfunding project with three statements (e.g., “Contributing to the project provides too much of a financial strain on me”). They are negatively related to donation behavior, and previous research on attitudes toward money finds that they assume importance when people are thinking about money (Furnham, 1984). Indeed, the most common reason given for not donating is not having enough money to spare. In the charitable crowdfunding context, people who are financially constrained have little ITD money, but are more willing to share a project on their own social network, because that is an important, yet costless, way to support a project (Li et al., 2017). Therefore, the following hypotheses are proposed: when controlling for the other motivations,

**H6a.** Financial constraints are positively related to individuals’ WTS.
**H6b.** Financial constraints are negatively related to individuals’ ITD.

### Social Interaction

#### Social Influence

Social influence is defined as people’s perception of how their close acquaintances believe they should consider a behavior (Li et al., 2017). We developed a combination of multiple scales such as beliefs in social norms and motivation to comply to, to measure social influence with four items (e.g., “I contribute to the project because my friends asked me to contribute”) (Jian and Shin, 2015). Individuals’ behavior is influenced by the preferences of their reference group and the social pressure they experience; this phenomenon is called “social influence” (Kulviwat et al., 2009). Previous literature has proved that friends’ support of a project and peer pressure can drive prosocial behavior (Barry and Wentzel, 2006). Such behavior is performed to comply with social norms and to improve one’s image in the community. Being a part of a social network whose approval is valued increases the perceived desirability of giving (Schervish and Havens, 1997). In the case of charitable crowdfunding, projects spread through SNSs. People extend their networks and maintain their social relationships through SNSs (Sura et al., 2017). Social pressure and social norms mean that they are more likely to be motivated to support charitable crowdfunding projects when being solicited by friends in their own social network. We can expect social influence, therefore, to play a specific role in shaping donors’ behaviors in charitable crowdfunding. Thus, the following hypotheses are proposed: when controlling for the other motivations,

**H7a.** Social influence is positively related to individuals’ WTS.
**H7b.** Social influence is positively related to individuals’ ITD.

#### Social Ties

Social ties refer to the social interaction between two or more individuals (Dong and Wang, 2018). Social ties between individuals in an online community are strengthened by familiarity and frequency of communication with each other (Zhang C.-B. et al., 2017). Following Liu et al. (2018)’s research, social tie was measured by the strength of the relationships, and the amount of time spent, and communication frequency among members of the charitable crowdfunding platforms with four items (e.g., “I spend a lot of time interacting with the project initiator on WeChat”). Strong social ties develop over time, and provide the basis of cooperation, trust, and collective actions in the online community (Zhang C.-B. et al., 2017). SNSs allow users to build social ties through communicating and sharing information, as in real-life connections (Sura et al., 2017). Charitable crowdfunding members can communicate with each other through personal messages and comments. Familiarity with the initiator of a project created by a brief dialogue increases the likelihood of contributions, possibly because familiarity increases liking and trust (Agrawal et al., 2015). People who are closer and more familiar with a project initiator are more inclined to support the project (Borst et al., 2018). We expect social ties between the initiator and individuals to have a positive effect on the individuals’ support for charitable crowdfunding. The specific assumptions are as follows: when controlling for the other motivations,

**H8a.** Social ties are positively related to individuals’ WTS.
**H8b.** Social ties are positively related to individuals’ ITD.

## Methodology

### Questionnaire Design and Construct Measurement

WeChat users were surveyed using a questionnaire to investigate the research questions. All the items on the questionnaire (Supplementary Table 2) were collected and adapted from validated measures used in the previous literature, with minor adjustments to fit the charitable crowdfunding context. Each item was measured using a seven-point Likert scale, ranging from 1 (“strongly disagree”) to 7 (“strongly agree”).

The questionnaire was originally developed in English and subsequently translated into Chinese. Then, three domain experts and four potential survey respondents were invited to complete the survey questionnaire and provided comments. Several minor changes were made as a result, and the Chinese version of the questionnaire was finalized.

We conducted a pre-test before the final data collection by sampling participants from a university located in eastern China. We received 60 valid responses, and the results showed that all the items satisfied the validity and reliability threshold values of the constructs.

### Data Collection

After finalizing the pilot study, an online survey was used to collect empirical data. Online commercial and professional online survey agencies WJX^1^ and CREDAMO^2^ were commissioned to recruit survey participants, targeting WeChat users via a personal invitation, from January 2019 to March 2020. To encourage participation, an incentive was provided; respondents who completed the questionnaire successfully received a payment of RMB 5 and an opportunity to win a coupon (worth a random amount between RMB 1 and 5) to be deposited into their WeChat wallet.

First, subjects were shown a brief introduction of charitable crowdfunding, survey purpose, and privacy protect of personal data, then they were asked to consent to participation in the study via a checkbox query; second, subjects were asked to recall the latest crowdfunding project they had read (regardless of whether they had donated money) and to provide the title of the project; finally, subjects were an instruction to fill out our questionnaire. The instruction reads, “The statements below are reasons that people may or may not want to help charitable crowdfunding initiators. Using the scale below, please indicate how much you agree with each of these statements in terms of how much it applies to you personally” (1 = strongly disagree, 7 = strongly agree). Then the subjects complete all the measures of different motives with self-rated points. In 7 months, a total of 1,100 (300 in WJX, 800 in CREDAMO) respondents were approached, of whom 819 (157 from WJX, 662 from CREDAMO) completed the questionnaire. We excluded 162 responses that did not specify a charitable crowdfunding project (each respondent was asked to fill in the title of the charitable crowdfunding project), and eliminated another 40 responses that were completed in an unrealistically short time or provided the same answers to all the questions. We ended up with 617 valid responses; a valid response rate of 56.1%.

Table 1 lists the demographic information of the respondents. To summarize, 51.9% were male and 48.1% were female; education levels varied from high school and lower, to graduate; and 67.2% had an income of more than RMB 5,000 per month.

**TABLE 1:** Demographic characteristics of the sample (*n* = 617).

Item	Category	Frequency	Ratio (%)
Gender	Male	320	51.9
	Female	297	48.1
Age	≤20	18	2.9
	21–30	298	48.3
	31–40	243	39.4
	41–50	51	8.3
	>50	7	1.1
Education	High school and less	142	23.1
	Junior college	277	44.9
	Undergraduate	171	27.7
	Graduate	27	4.4
Personal income	≤CNY 2000	52	8.4
per month	CNY 2001–5000	151	24.5
	CNY 5001–8000	246	39.9
	CNY 8001–15000	130	21.1
	>CNY 15000	38	6.2
Past donation	Never (0 time)	22	3.6
experience	Seldom (1–3 times)	225	36.5
	Sometimes (4–5 times)	206	33.4
	Frequently (more the 5 times)	164	26.6

## Results

This study used a three-step approach to analyze the research model. The first step involved the analysis of the measurement model, where reliability, validity, and common method bias (CMB) were examined. The second step tested the effects of independent and control variables on two dependent variables separately, using hierarchical ordinary least squares (OLS) regression. In the third step, we delineated the structural model, which links all explanatory variables that had significant relations with the two dependent variables based on the results of the regression analysis. IBM SPSS Statistics 20 and SmartPLS 2.0 were used to analyze the data.

### Measurement Model

#### Common Method Bias

As all the data were self-reported from a single source, CMB arising from consistency motif and social desirability could be a problem (Podsakoff and Organ, 1986). To eliminate CMB, we carefully designed the survey so that simple and specific questions were asked. Multiple questions were set up for each concept. Moreover, the Harman one-factor test was conducted to examine whether the majority of the variance could be accounted for by one factor (Podsakoff et al., 2003). The analysis results show that all the items can be categorized into 10 factors, and that the most covariance accounted for by any one factor is only 15.097%. This suggests that CMB is not a serious concern in this analysis.

#### Reliability, Convergent Validity, and Discriminant Validity

Reliability was assessed by examining the constructs’ Cronbach’s α, composite reliability (CR), and average variance extracted (AVE). As Table 2 shows, Cronbach’s α and CR values of all constructs exceed the recommended threshold of 0.7, and the AVE values exceed the recommended threshold of 0.5 (Fornell and Larcker, 1981). *Convergent validity* was evaluated by considering the loadings of all indicators. All the loading scores shown in Table 2 are above the benchmark level of 0.7 (Hair et al., 2014). *Discriminant validity* was assessed in two steps. First, we conducted an exploratory factor analysis (EFA) using IBM SPSS Statistics 20. Rotated component loading clearly indicated that all indicators’ loadings on the associated constructs are greater than all their loadings on other constructs. Second, an AVE analysis was conducted. As shown in Table 3, all the square root values of the AVE (values on the diagonal) exceed the inter-construct correlations, suggesting good discriminant validity (Fornell and Larcker, 1981).

**TABLE 2:** Construct reliability and convergent validity (*n* = 617).

Constructs	Items	Mean	SD	Factor loading	Mean (STD)	Cronbach’s α	CR	AVE
Reputation	REPU1	4.21	1.891	0.815	4.35 (1.07)	0.956	0.964	0.791
	REPU2	4.56	1.876	0.877				
	REPU3	4.63	1.924	0.861				
	REPU4	4.33	1.976	0.887				
	REPU5	4.35	1.935	0.872				
	REPU6	4.46	1.881	0.798				
	REPU7	3.88	1.919	0.718				
Reciprocity	RECP1	5.72	1.275	0.828	5.78 (1.05)	0.855	0.909	0.769
	RECP2	5.80	1.112	0.731				
	RECP3	5.81	1.192	0.843				
Sense of belonging	SOBE1	5.70	1.214	0.753	5.78 (1.05)	0.914	0.939	0.795
	SOBE2	5.71	1.233	0.822				
	SOBE3	5.82	1.172	0.837				
	SOBE4	5.88	1.114	0.817				
Joy of giving	JOGI1	5.24	1.577	0.745	5.39 (1.29)	0.892	0.925	0.755
	JOGI2	5.39	1.462	0.728				
	JOGI3	5.28	1.536	0.753				
	JOGI4	5.65	1.345	0.697				
Altruism	ALTR1	5.64	1.190	0.792	5.69 (0.98)	0.900	0.926	0.715
	ALTR2	5.56	1.170	0.793				
	ALTR3	5.67	1.132	0.765				
	ALTR4	5.63	1.213	0.733				
	ALTR5	5.94	1.060	0.719				
Financial constraints	CONT1	3.63	1.900	0.913	3.50 (1.79)	0.933	0.957	0.882
	CONT2	3.59	1.875	0.921				
	CONT3	3.28	1.930	0.889				
Social influence	SOIN1	5.00	1.535	0.705	5.17 (1.37)	0.917	0.941	0.801
	SOIN2	5.29	1.452	0.816				
	SOIN3	5.09	1.626	0.783				
	SOIN4	5.29	1.498	0.793				
Social tie	STIE1	4.36	1.890	0.853	4.10 (1.84)	0.955	0.967	0.880
	STIE2	4.09	1.948	0.850				
	STIE3	3.94	1.980	0.866				
	STIE4	3.98	2.013	0.872				
Willingness to share	WTS1	4.81	1.740	0.834	4.80 (1.73)	0.973	0.982	0.948
	WTS2	4.74	1.780	0.835				
	WTS3	4.85	1.800	0.836				
Intention to donate	ITD1	5.59	1.184	0.741	5.59 (1.14)	0.942	0.963	0.896
	ITD2	5.62	1.173	0.737				
	ITD3	5.56	1.242	0.742				

*The results of the aforementioned tests not only prove that the survey scales are reliable and valid but also demonstrate that the distinction between different motivations indeed exists, both theoretically and empirically.*

**TABLE 3:** Correlations among research variables.

Variables	1	2	3	4	5	6	7	8	9	10
1. Reputation	0.889									
2. Reciprocity	0.333**	0.877								
3. Sense of belonging	0.164**	0.473**	0.892							
4. Joy of giving	0.457**	0.392**	0.512**	0.869						
5. Altruism	0.304**	0.434**	0.534**	0.575**	0.846					
6. Financial constraints	0.295**	−0.005	−0.178**	0.091*	0.010	0.939				
7. Social influence	0.579**	0.347**	0.336**	0.494**	0.383**	0.127**	0.895			
8. Social Tie	0.454**	0.101*	0.158**	0.398**	0.263**	0.464**	0.186**	0.938		
9. Willingness to share	0.409**	0.261**	0.361**	0.506**	0.489**	0.455**	0.266**	0.529**	0.974	
10. Intention to donate	0.292**	0.412**	0.557**	0.545**	0.690**	0.396**	0.396**	0.329**	0.547**	0.947

*p < 0.05, **p < 0.01. Diagonal italic values are the square roots of AVE of each construct.

### Regression Model

#### Regression Model Setting

We first used hierarchical OLS regression to test the effects of independent variables on the two dependent variables separately. As the dependent variables, WTS and ITD, are continuous, we use OLS regression for the multivariate analyses.

First, the extrinsic and intrinsic motivations were entered into regression. The model is expressed as follows:

(1) $$\begin{array}{cc} Y=\alpha_{i}+\beta_{1}\times \mathrm{Reputation}+\beta_{2}\times \mathrm{Reciprocity}+\beta_{3}\times & \\ \mathrm{Sense}\mathrm{of}\mathrm{belonging}+\beta_{4}\times \mathrm{Joy}\mathrm{of}\mathrm{giving}+\beta_{5}\times & \\ \mathrm{Altruism}+\beta_{6}\times \mathrm{Financial}\,\mathrm{constraints}+\mu & \end{array}$$

Second, social interaction motivations were entered into the regression. The model is expressed as follows:

(2) $$\begin{array}{cc} Y=\alpha_{i}+\beta_{1}\times \mathrm{Reputation}+\beta_{2}\times \mathrm{Reciprocity}+\beta_{3}\times & \\ \mathrm{Sense}\,\mathrm{of}\,\mathrm{belonging}+\beta_{4}\times \mathrm{Joy}\,\mathrm{of}\,\mathrm{giving}+\beta_{5}\times \mathrm{Altruism}+ & \\ \beta_{6}\times \mathrm{Financial}\,\mathrm{constraints}+\beta_{7}\times \mathrm{Social}\,\mathrm{influence}+ & \\ \beta_{8}\times \mathrm{Social}\,\mathrm{tie}\,\mathrm{with}\,\mathrm{the}\,\mathrm{initiator}+\mu & \end{array}$$

In Eqs 1, 2,_α_i_ is the intercept and β_1_ are the corresponding coefficients for the independent variables. The error term of the model is denoted by μ. The dependent variable is shown as *Y*, corresponding to WTS and ITD.

### Multiple Regression Analyses

Before the regression analyses were performed, we checked for multicollinearity. The tolerance values for all explanatory variables ranged from 0.492 to 0.854, well above the cut-off value of 0.1, and the variance inflation factor values of these variables ranged from 1.232 to 2.023, well below the cut-off value of 10 (Hair et al., 1995). Hence, multicollinearity was not a serious problem.

Ordinary least squares estimates of regression coefficients are shown in Table 4. The last five columns present diagnostic statistics: *R*^2^, adjusted *R*^2^, the Durbin–Watson statistics, model *F*, and significance of model *F*. Since the Durbin–Watson statistics are near 2.0, ranging from 1.963 to 1.990 for all models, there is little serial correlation in the residuals.

**TABLE 4:** Regression predicting WTS and ITD.

	Willingness to share		Intention to donate	
	Model 1	Mode 2	Model 3	Model 4
Reputation	0.222***	0.078*	0.037*	−0.003
Reciprocity	−0.106	−0.030	0.045	0.067**
Sense of belonging	0.178**	0.129*	0.211***	0.197***
Joy of giving	0.292***	0.170***	0.119***	0.085**
Altruism	0.473***	0.422***	0.551***	0.527***
Financial constraints	0.042	−0.024	−0.035*	−0.054**
Social influence		0.104**		0.028
Social tie		0.319***		0.090***
Constant	−0.992**	−1.191***	0.293	0.238
DW	1.972	1.990	1.963	1.981
*R*^2^	0.357	0.450	0.546	0.563
Adjusted *R*^2^	0.351	0.443	0.542	0.558
*F*	56.537	62.182	122.428	98.080
Sig	0.000	0.000	0.000	0.000

n = 617; *p < 0.10, **p < 0.05, and ***p < 0.01.

Inclusion of extrinsic and intrinsic motivations in model 1 accounted for a significant proportion of the variance in WTS: adjusted *R*^2^ = 0.357, *F* = 56.537, *p* < 0.001. As expected, reputation (β = 0.222, *p* < 0.01), sense of belonging (β = 0.178, *p* < 0.05), joy of giving (β = 0.292, *p* < 0.01), and altruism (β = 0.473, *p* < 0.01) predicted WTS. Reciprocity and financial constraints were not shown to predict WTS. These results provided strong support for H1a, H3a, H4a, and H5a. In model 2, inclusion of social interaction motivations moderately increased the variance explained (adjusted *R*^2^ = 0.450, *F* = 62.182, *p* < 0.001), and the explanatory power of the model increased by 9.3%. Inspection of the beta weights revealed significant effects for both social influence (β = 0.104, *p* < 0.05) and social ties (β = 0.319, *p* < 0.01). The results provided strong support for H7a and H8a. The significance level of beta weights changed for reputation and sense of belonging. The effects of reputation and sense of giving were still significant, but only at the *p* < 0.10 level, which indicates that, considering social interaction, people were less concerned about their reputation and sense of belonging.

Inclusion of extrinsic and intrinsic motivations in model 3 accounted for a significant amount of the variance in ITD (adjusted *R*^2^ = 0.542, *F* = 122.428, *p* < 0.001). As expected, reputation (β = 0.037, *p* < 0.1), sense of belonging (β = 0.211, *p* < 0.1), joy of giving (β = 0.119, *p* < 0.01), altruism (β = 0.551, *p* < 0.01), and financial constraints (β = −0.035, *p* < 0.1) predicted ITD. The results provide support for H1b, H3b, H4b, H5b, and H6b. In model 4, inclusion of social interaction motivations moderately increased variance explained (adjusted *R*^2^ = 0.558, *F* = 98.080, *p* < 0.001), and the explanatory power of the model increased by 1.6%. The beta weights revealed a significant effect only for social ties (β = 0.090, *p* < 0.01). This result provides strong support for H8b. The significance level of beta weights changed for reciprocity and financial constraints. The beta weights reveal significant effects for both reciprocity (β = 0.067, *p* < 0.05) and financial constraints (β = −0.054, *p* < 0.05). The results indicate that, considering social interaction, stronger social ties were built, and individuals’ ITD money to the project was positively affected by their expectation for reciprocity and negatively affected by financial constraints.

The hypotheses testing results are summarized in Table 5. In conclusion, 12 out of the 16 hypotheses are supported, and four hypotheses are not supported.

**TABLE 5:** Summary of regression results.

Hypotheses	Independent variables	Willingness to share	Results	Intention to donate	Results
Extrinsic motivation	Reputation	H1a: β = 0.078, *p* < 0.1	Support	H1b: β = −0.003, *p* < 0.1	Not Support
	Reciprocity	H2a: β = −0.030, *p* < 0.1	Not support	H2b: β = 0.067, *p* < 0.05	Support
Intrinsic motivation	Sense of belonging	H3a: β = 0.129, *p* < 0.1	Support	H3b: β = 0.197, *p* < 0.01	Support
	Joy of giving	H4a: β = 0.170, *p* < 0.01	Support	H4b: β = 0.085, *p* < 0.05	Support
	Altruism	H5a: β = 0.422, *p* < 0.01	Support	H5b: β = 0.527, *p* < 0.01	Support
	Financial constraints	H6a: β = −0.024, *p* < 0.1	Not support	H6b: β = −0.054, *p* < 0.05	Support
Social interaction	Social influence	H7a: β = 0.104, *p* < 0.05	Support	H7b: β = 0.029, *p* < 0.1	Not support
	Social tie	H8a: β = 0.39, *p* < 0.01	Support	H8b: β = 0.090, *p* < 0.01	Support

### Structural Model

**FIGURE 2:**
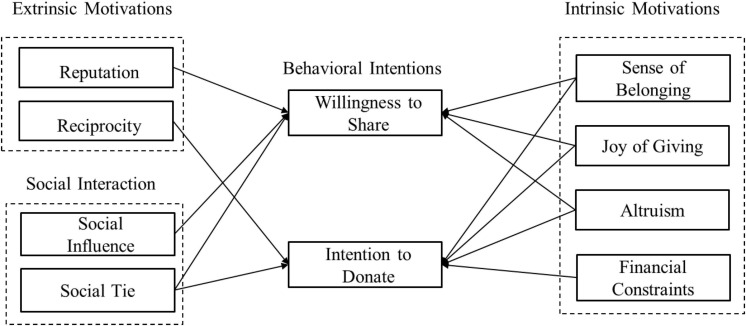
Confirmatory model of motivations behind donors’ behavior intention.

**FIGURE 3:**
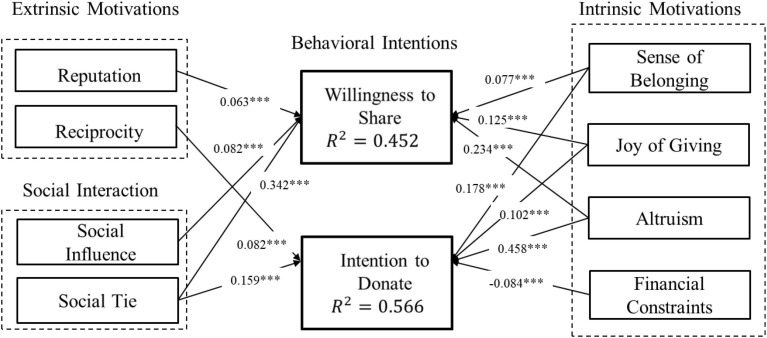
Results of structural model. ***p < 0.001.

Based on the results of the regression analysis, we depict the structural model, which links all significant explanatory variables with the two dependent variables (i.e., WTS and ITD). Figure 2 presents the confirmatory structural model of factors predicting charitable crowdfunding behavior. Figure 3 presents the parameter estimates of the structural model.

As illustrated in Figure 3, three explanatory motivation variables (i.e., sense of belonging, joy of giving, and altruism) display a significant positive effect on improvement in charitable crowdfunding behavioral intentions (i.e., WTS and ITD). The extrinsic motivations, reputation and reciprocity, each display a significant positive effect only on WTS and ITD, respectively. The intrinsic motivation variable, financial constraints, displays a significant negative effect only on ITD. In addition, the social connection variable–social ties–is associated positively with both WTS and ITD. However, social influence displays a significant positive effect only on WTS.

In summary, all hypotheses receive strong support from both the regression analysis and the structural model. As shown in Table 5 and Figure 3, the results of these two analytical approaches are consistent in both their estimates and the significance levels of their coefficients. This provides confidence in the robustness of our findings.

## Conclusion

The aim of this study was to investigate the psychological and social motivations underlying decisions to support charitable crowdfunding projects by testing the effectiveness of self-determination theory and the social interaction model with self-reported data.

With regard to individuals’ WTS project information, three intrinsic motivations–sense of belonging, joy of giving, and altruism–emerge as the strongest self-reported motivations. These results confirm the suggestions by Burtch et al. (2013), Choy and Schlagwein (2016), and Cecere et al. (2017), respectively. One extrinsic motivation–reputation–appears to be a significant factor explaining WTS project information. The two social interaction motivations (i.e., social influence and social ties) are significant factors determining individuals’ WTS project information. These results are consistent with the findings of Cecere et al. (2017). The extrinsic motivation variable, reciprocity, is not found to be related to WTS project information, which can be explained by the fact that sharing project information with one’s own social network is a costless way to support a project, and donations are made by crowds anonymously; thus, reciprocity is not expected on social networks.

With regard to individuals’ ITD money to charitable crowdfunding projects, three intrinsic motivations–sense of belonging, joy of giving, and altruism–also emerge as the strongest self-reported motivations. The intrinsic motivation–financial constraints–is significantly, negatively, related to ITD. These results are consistent with the previous studies of Choy and Schlagwein (2016), Cecere et al. (2017), and Konrath and Handy (2018). One extrinsic motivation variable–reciprocity–appears to be a significant factor in explaining ITD money in charitable crowdfunding. As for social interaction motivations, only social ties shows significant effects on ITD, which is consistent with the findings on reward-based crowdfunding of Liang and Yuan (2016). Contrary to our expectations, reputation concern and social influence do not have a significant impact on the ITD. This might be due to the fact that many charitable donations on crowdfunding platforms are given anonymously, and that people’s concern for their reputation in social networks is limited; therefore, it seems unlikely that a contributor could gain much in the way of reputation from making a contribution. For similar reasons, their close acquaintances would be unable to recognize their charity decisions unless they chose to display their donations on their own social network; thus, social influence might not be a concern for people’s donation decisions in the crowdfunding context (Gleasure and Feller, 2016).

In addition, the research findings indicate that the motivators for individuals to share project information and donate money to a project are the same in some aspects but differ in others.

First, intrinsic factors, including sense of belonging, joy of giving, and altruism, are factors that explain people’s support (both WTS and ITD). These results indicate that people’s support for charitable crowdfunding projects yields mainly psychological benefits for the supporters. The results also confirm previous findings in the traditional charity context, that support is, in many cases, an almost automatic emotional response, producing a positive mood, and making supporters feel themselves to be morally just (Bekkers and Wiepking, 2011).

Second, social ties turn out to be another factor that explains both charitable crowdfunding behaviors, especially WTS. Inclusion of social connection variables increases the explanatory power of the model by 8.4% for WTS, but by only 1.4% for ITD, which indicates that social interaction motivations explain more additional variance in WTS. These findings indicate that communication and interaction with a project initiator creates strong social ties, which increases people’s intention to support the project. This mechanism can be explained by the possible mediator of trust in the initiator; social interaction increases supporters’ trust in the initiator, then increases their intention to support the initiator (Liu et al., 2018).

Third, reputation concern and social influence display significant positive effects on WTS, but not on ITD. These results indicate that sharing charitable crowdfunding projects with one’s own social network enables one to gain recognition. People’s sharing behavior is also influenced by the behavior of their friends on their social networks. These results can be explained by the fact that support for charitable crowdfunding can be recognized by the public only when people choose to share the project through their own social networks. People’s identities are known in their own social network, their actions are observable, and they perceive those observing them as important; they are more likely, then, to be concerned about how their actions will be viewed (Burtch et al., 2013). People who are willing to share charitable crowdfunding project information are motivated by reputational gains that they might make from being involved in charity. Similarly, people who are influenced by their friends in social networks also have the motivation to show their support for projects by, for example, sharing project information with their friends on social networks.

Fourth, reciprocity and financial constraints are predictors of ITD, but are not predictors of WTS. As expected, financial constraints negatively influence donations. Reciprocity is the power to enhance collective actions and enforce social norms. People anticipate enhancing cooperative behavior in online social communities by donating money to charitable crowdfunding; they then expect others to support charitable crowdfunding and generate more donations in the future.

### Implications

Charitable crowdfunding is different from traditional charity because of its social network characteristics. Although factors influencing decisions to make donations to traditional charities have been studied widely, the topic has seldom been discussed in the context of charitable crowdfunding. The current study fills this knowledge gap. It finds that project success is determined not only by individuals’ personal donations but also by their sharing behaviors, which will, in turn, impact project funding. To our knowledge, this is one of the first studies to focus on individuals’ WTS in charitable crowdfunding. Furthermore, this study highlights the importance of considering multi-dimensional motivations in charitable crowdfunding behavior. It examines the direct effects of intrinsic and extrinsic motivations and social interaction motivations, providing a comprehensive understanding of individuals’ charitable crowdfunding behaviors. It also investigates the differences in motivations influencing WTS and ITD.

This study has a number of important implications for both charitable crowdfunding platforms and fundraisers. Concerning intrinsic motivations, the results suggest that sense of belonging, joy of giving, and altruism serve as crucial motivators in supporting charitable giving behaviors. Charitable crowdfunding platforms could offer ways of boosting users’ sense of belonging, such as by adding social networking features. Updates about how the donations are helping the project initiator could also be provided to increase donors’ experienced joy of giving. Promoting individuals’ awareness of altruism, through publicity and education, could significantly improve the crowd’s involvement. The extrinsic motivations, reputation and reciprocity, contribute to WTS and ITD, respectively. This suggests that increasing numbers of active members of the charitable crowdfunding platforms will strengthen the opportunities for communication and participation, thus increasing each individual’s acquaintances, and the level of social community interaction. As for social interaction motivations, social ties are crucial determinants of supporting behaviors. This result suggests that project initiators should make an effort to build social ties with supporters and to keep their projects active through continued updates and postings. Social influence also plays a role in motivating sharing behaviors. The initiator could spread awareness of the project to his or her close acquaintances, such as friends and family, to broadcast the project to a broader audience.

### Limitations

This study has certain limitations. First, self-reported motives and charitable crowdfunding behavioral intentions may be inaccurate; people are inclined to present themselves in a more favorable light and may have reported misleadingly high evaluations of their intrinsic motivations, low evaluations of their extrinsic motivations, and high level of their intentions to donate. However, we used multiple items to assess each construct to address this potential issue, and assured participants of the anonymity and confidentiality of their responses. Second, the dataset we analyzed was cross-sectional, and unobservable heterogeneity among the analyzed factors might be present: longitudinal analyses are therefore recommended for future research. Third, the samples in this research were collected from China, and through only one SNS: the WeChat channel. Future research could employ more extensive sampling in countries that vary in culture and in multiple SNSs. Fourth, more complex social and psychological mechanisms could be examined to explain charitable crowdfunding behaviors, especially the antecedents of various motivations (e.g., anticipated guilt if not helping, a more complex motive for helping that may work as mediator and moderator between other motives and behavioral intentions), and how those antecedents and motivations influence behaviors. Fifth, measurements of the motives can be operationalized in multiple ways and different operationalizations can lead to different results. Items used in this research were specifically adapted to charitable crowdfunding context and could serve as alternative measurement for motivations and intentions. Further research on different types and operationalizations of motives and intentions might draw different results.

## Data Availability Statement

The raw data supporting the conclusions of this article will be made available by the authors, without undue reservation.

## Ethics Statement

Ethical review and approval was not required for the study on human participants in accordance with the local legislation and institutional requirements. The participants provided their written informed consent to participate in this study.

## Author Contributions

All authors together conceptualized the study and approved the final version of the article for submission. HJ designed the study, collected data, and performed analysis under the supervision of LM and HJ, interpreted the results, and drafted the manuscript. LQ, TL, and LM contributed with interpretations and revisions of the manuscript draft.

## Conflict of Interest

The authors declare that the research was conducted in the absence of any commercial or financial relationships that could be construed as a potential conflict of interest.

## Footnotes

1 www.wjx.cn

2 www.credamo.com
