# Supplementary material for: Why Do People Support Online Crowdfunding Charities? A Case Study From China
Source: Front Psychol. 2021 Feb 26;12:582508. doi: 10.3389/fpsyg.2021.582508 (PMC7952437; doi:10.3389/fpsyg.2021.582508)
Supplement: Supplementary file 1 [file Data_Sheet_1.docx]

**Appendix**

The pictures below have shown a specific medical crowdfunding project in Shuidichou platform, and this project was spread in WeChat. Generally, supplicants launch projects through Shuidichou platform, fill in personal information, target amount, help instructions and other detailed information. WeChat users have access to fundraising projects through the “love home page” column of Shuidichou Official Accounts, personal Moments and private messages sent by friends. Browsing the project details, users can forward and make donations according to their personal wishes.

**Supplementary Figure 1** The WeChat page of a medical crowdfunding project on Shuidichou


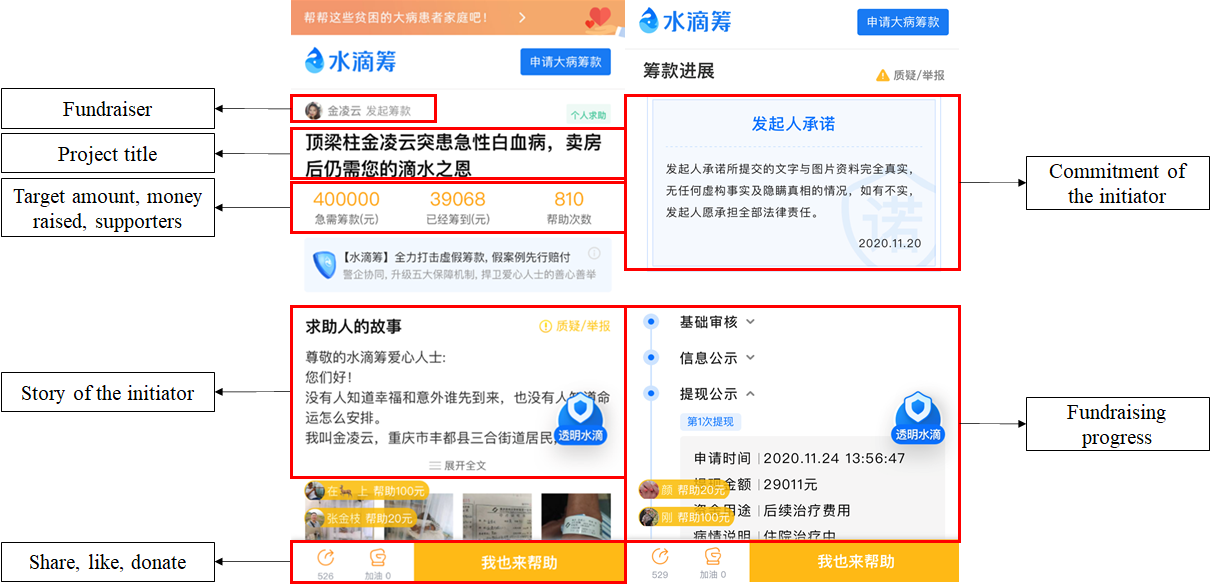


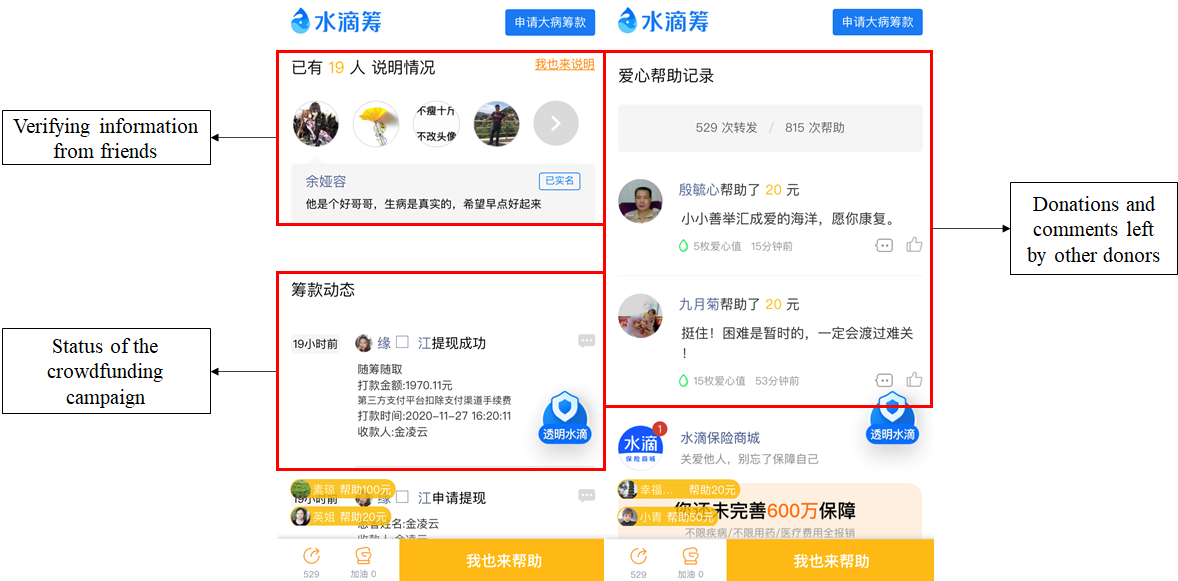


**Supplementary Table 1** Quantitative studies on factors influencing behavioural intention in charitable crowdfunding

| No | Reference | Factors | Intention to donate-related variables |
| --- | --- | --- | --- |
| 1 | Sura et al. (2017) | Charity Project; Charity Organization; Internet Technology Features; SNS Features;  General attitude toward online donations (+) | Intention to Donate |
| 2 | Li et al. (2018) | Performance Expectancy (+)  Effort Expectancy (+)  Social Influence (+)  Facilitating Conditions (+)  Sense of Trust (+)  Experience Expectation (+) | Intention to Donate |
| 3 | Liu et al. (2018) | Website Quality, Transaction Convenience; Initiator Reputation; Project Popularity;  Project Content Quality  Empathy (+)  Perceived Credibility (+) | Intention to Donate |
| 4 | Wang et al. (2019) | Sense of Self-worth; Face Concern; Perceived Donor Effectiveness; Moral Obligation; Social Interaction; Referent Network Size  Self-identity (+)  Social identity (+) | Donation intention |
| 5 | Chen et al. (2019) | Social Presence (+) (+)  Trust (+) (+)  Attitude (ns) (+)  Subjective Norms(ns) (ns)  Perceived Behavioural Control (+) (+)  Personal Norms (+) (+) | Time Donations; Money Donations |

Note: Relationship between factors and intention to donate variables (ns: not significant; +: positive).

**Supplementary Table 2** Constructs and Scale Items

| Construct |  | Measurement items | References |
| --- | --- | --- | --- |
| Reputation | REPU1 | I wish to publicly show my support to the project | Choy & Schlagwein, 2016; Konrath & Handy, 2018; Jian & Shin, 2015 |
|  | REPU2 | I want my friends/social interactions to have a good impression on me |  |
|  | REPU3 | I expected to receive attention from my friends/social interactions consciously |  |
|  | REPU4 | I expected to receive recognition from my friends/social interactions consciously |  |
|  | REPU5 | I expected to receive credibility for my friends/social interactions consciously |  |
|  | REPU6 | I wish to signal a certain image of myself to others |  |
|  | REPU7 | I have the prospect of receiving rewards for my contribution |  |
| Reciprocity | RECP1 | I expect that others will help me when I am in need for my contribution | Zhang et al., 2017; Hung et al., 2011 |
|  | RECP2 | I believe that others will help me when I am in need for my contribution |  |
|  | RECP3 | I expect that my requirements will be fulfilled when I am in need |  |
| Sense of Belonging | SOBE1 | I am a “part of the project” together with “like-minded people” | Choy & Schlagwein, 2016; Wang et al., 2019 |
|  | SOBE2 | I feel of belonging to a positive group/team or community |  |
|  | SOBE3 | I share the aim of supporting the project with other donors |  |
|  | SOBE4 | I do meaningful contribution with other donors to the aim of supporting the project |  |
| Joy of Giving | JOGI1 | Contributing to the project makes me feel powerful | Choy & Schlagwein, 2016; Konrath & Handy, 2018 |
|  | JOGI2 | Contributing to the project makes me feel needed |  |
|  | JOGI3 | Contributing to the project makes me feel self-satisfied |  |
|  | JOGI4 | Contributing to the project makes me feel happy |  |
| Altruism | ALTR1 | I like helping other people even though it is not required | Liu et al, 2018; Konrath & Handy, 2018 |
|  | ALTR2 | I am always ready to help others |  |
|  | ALTR3 | I am willing to give my time to help others |  |
|  | ALTR4 | I give because I am concerned about those less fortunate than myself |  |
|  | ALTR5 | I donate because I feel compassion toward people in need |  |
| Financial Constraints | CONT1 | Contributing to the project would interfere with me meeting my own financial obligations | Konrath & Handy, 2018 |
|  | CONT2 | Even if I wanted to contribute, I could not financially afford it |  |
|  | CONT3 | Contributing to the project provides too much of a financial strain on me |  |
| Social Influence | SOIN1 | I contribute to the project because my friends asked me to contribute | Jian & Shin, 2015 |
|  | SOIN2 | Because my friends contribute |  |
|  | SOIN3 | My friend’s donation played a role in my decision to contribute |  |
|  | SOIN4 | Other people who have connection with me contribute |  |
| Social Tie | STIE1 | I maintain close social relationships with the project initiator on WeChat | Liu et al., 2018 |
|  | STIE2 | I spend a lot of time interacting with the project initiator on WeChat |  |
|  | STIE3 | I know the project initiator on a personal level |  |
|  | STIE4 | I have frequent communication with the project initiator on WeChat |  |
| Willingness to Share | WTS1 | I would share this project on my social accounts (WeChat/Weibo) | Li et al., 2017 |
|  | WTS2 | I would recommend this project to my friends or family |  |
|  | WTS3 | I would tweet about this project with my own comments |  |
| Intention to Donate | ITD1 | The probability that I would donate money to the crowdfunding project is high | Liu et al., 2018 |
|  | ITD2 | My willingness to donate money to the crowdfunding project is high |  |
|  | ITD3 | The likelihood of my donating money to the crowdfunding project is high |  |
